# BiRank: Fast and Flexible Ranking on Bipartite Networks with R and Python

**DOI:** 10.21105/joss.02315

**Published:** 2020-07-10

**Authors:** Kai-Cheng Yang, Brian Aronson, Yong-Yeol Ahn

**Affiliations:** 1 Luddy School of Informatics, Computing, and Engineering, Indiana University, Bloomington, IN; 2 Department of Sociology, Indiana University, Bloomington, IN

## Summary

**Figure 1:**
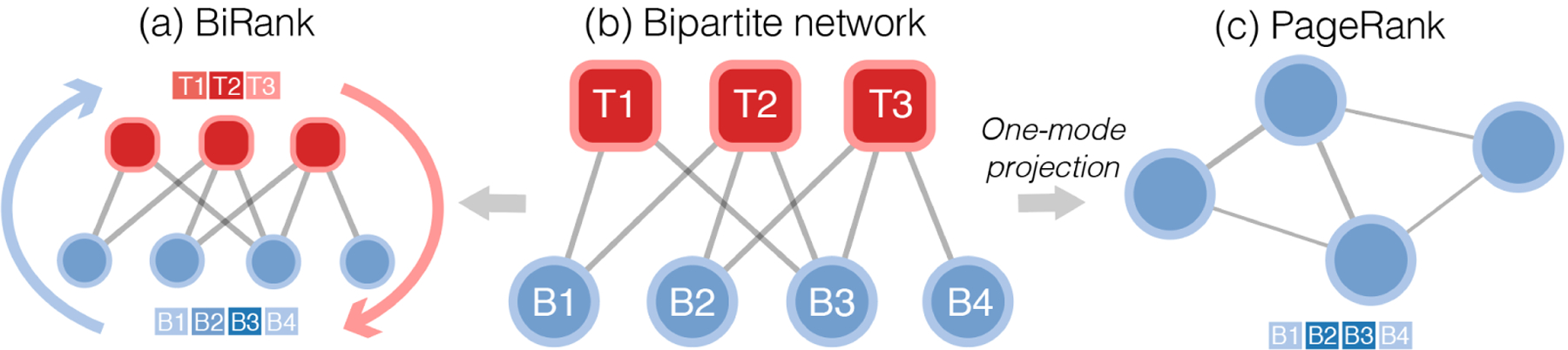
(a) BiRank algorithms perform the ranking process on the bipartite networks directly and generate the ranking values for the top and bottom nodes simultaneously. (b) A bipartite network with three top nodes and four bottom nodes. (c) After the one-mode projection, a unipartite network of the bottom nodes is generated. PageRank can be performed to generate the ranking values of the bottom nodes.

Bipartite (two-mode) networks are ubiquitous. Common examples include networks of collaboration between scientists and their shared papers, networks of affiliation between corporate directors and board members, networks of patients and their doctors, and networks of competition between companies and their shared consumers. Bipartite networks are commonly reduced to unipartite networks for further analysis, such as calculating node centrality (e.g. PageRank, see Figure 1(c)). However, one-mode projections often destroy important structural information (Lehmann, Schwartz, & Hansen, 2008) and can lead to imprecise network measurements. Moreover, there are numerous ways to obtain unipartite networks from a bipartite network, each of which has different characteristics and idiosyncrasies (Bass et al., 2013).

To overcome the issues of one-mode projection, we present BiRank, an R and Python package that performs PageRank on bipartite networks directly. The BiRank package contains several ranking algorithms that generalize PageRank to bipartite networks by propagating the probability mass (or importance scores) across two sides of the networks repeatedly using the following equations:

$$T=\alpha S_{T}B+(1-\alpha )T^{0}$$

$$B=\beta S_{B}T+(1-\beta )B^{0}$$

until they converge (see Figure 1(a)), where **T**, **B** are the ranking values for the top and bottom nodes, elements in **T**^0^ and **B**^0^ are set to 1/|**T**| and 1/|**B**| by default, *α* and *β* are damping factors and set to 0.85 by default, *S*_*T*_, *S*_*B*_ are the transition matrices. Unlike the one-mode projected PageRank, BiRank algorithms generate the ranking values for nodes from both sides simultaneously and take account of the full network topology without any information loss.

Our package implements the most notable and straightforward operationalizations of biparitite PageRanks including HITS (Kleinberg, 1999; Liao, Xiao, Cimini, & Medo, 2014), CoHITS (Deng, Lyu, & King, 2009), BGRM (Rui, Li, Li, Ma, & Yu, 2007), and Birank (He, Gao, Kan, & Wang, 2016). The algorithms mainly differ in the way they normalize node ranks in the iterations (see Table 1).

Our guiding philosophy is to make the package as flexible as possible, given the diverse array of problems and data formats that are used in network analysis, while achieving good performance. We therefore provide a number of convenience options for incorporating edge weights into rank estimations, estimating ranks on different types of input (edge lists, dense matrices, and sparse matrices), multiple file formats (as vectors, lists, or data frames), and for estimating PageRank on the one-mode projection of a network. Moreover, this implementation uses efficient data storage and algorithms to ensure good performance and scalability. For example, regardless of the algorithm of choice, it takes less than 10 seconds and less than 1GB of RAM to estimate ranks on a bipartite network containing half a million top nodes, more than two million bottom nodes, and about three million edges on a machine with 16 AMD EPYC 7000 series 2.5 GHz processors.

**Figure 2:**
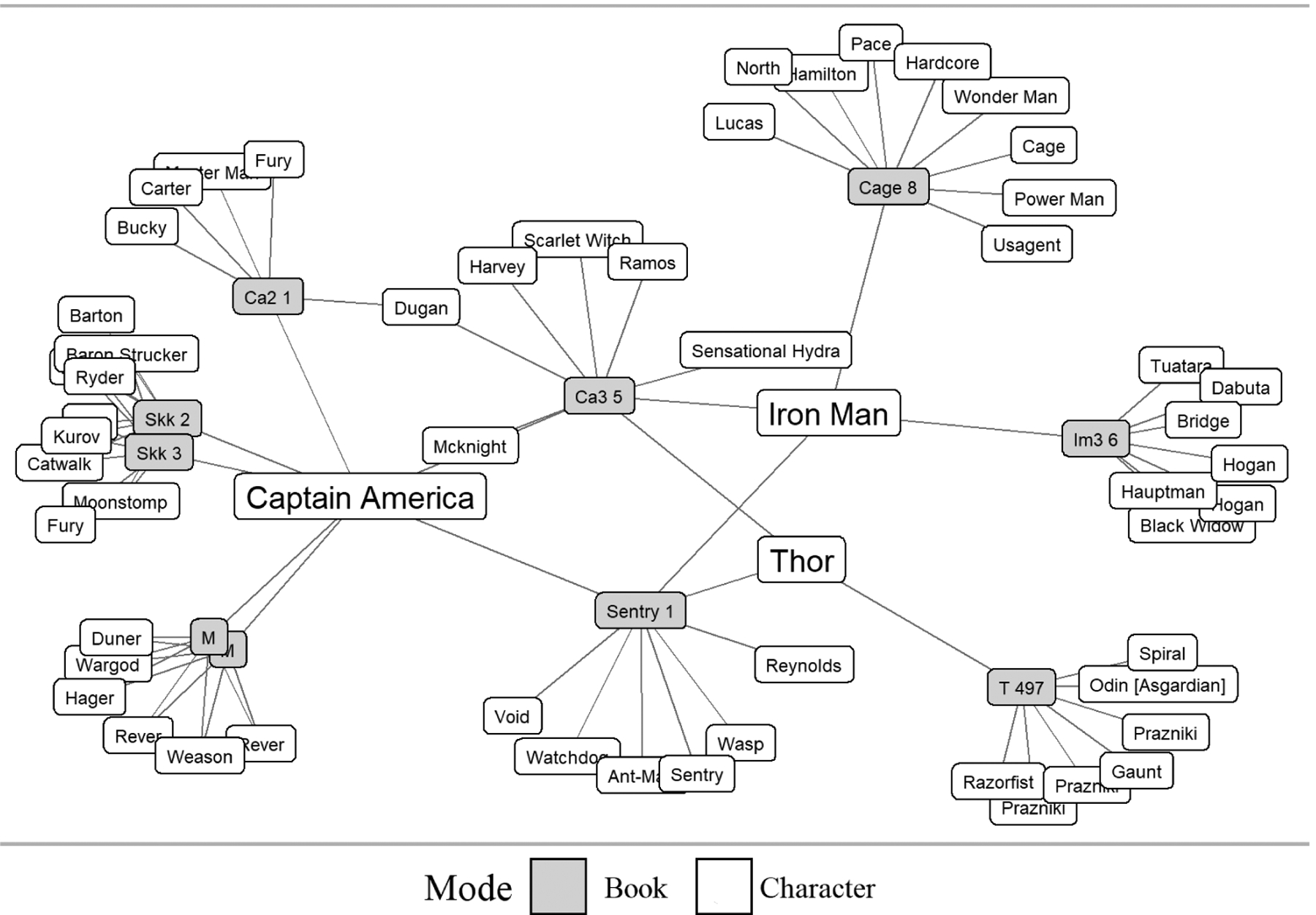
Sociogram of character-book ties within 10 comic books of the Marvel Universe collaboration network.

As a demonstration, we apply HITS, CoHITS, and one-mode projected PageRank to the Marvel Universe collaboration network (Alberich, Miro-Julia, & Rosselló, 2002). The Marvel Universe collaboration network comprises a network of affiliation with ties between every Marvel comic book (n = 12,849) and every character (n = 6,444) who appeared in those books. To give a sense of this network’s structure, Figure 2 illustrates a small sociogram of characters within ten comic books of this dataset.

Table 2 presents the five characters with the highest ranking values from each algorithm. Results are similar, with Captain America and Iron Man occurring in all three ranking algorithms. However, discrepancies arise from differences in the underlying ranking algorithms and how they interact with the network’s structure. PageRank on the one mode projection first converts comic-character ties to character-character ties. Without information about the structure of characters-comic ties, PageRank mainly prioritizes nodes with a large number of transitive ties in the original network. For example, Wolverine has a higher PageRank than the Thing but Wolverine appears in much fewer comic books than the Thing. Instead, Wolverine’s high PageRank is a result of his co-presence in comic books with large numbers of other characters. In contrast, the Thing tends to repeatedly appear in central comic books with other central characters in the Marvel universe, hence the Thing has a high CoHITS rank but a lower PageRank than Wolverine.

Differences between how HITS and CoHITS estimate ranks on the Marvel Universe collaboration network are more complicated. CoHITS normalizes the transition matrix by the outdegree of the source nodes, and therefore places somewhat less value on connections from highly connected characters and from highly connected comic books than HITS. As a result, CoHITS tends to assign higher ranks to characters who are connected to a more diverse array of comic books than does HITS. This difference is best illustrated by the inclusion of Mr. Fantastic in HITS’ top-ranked characters and the inclusion of Spider Man in CoHITS’ top-ranked characters: Spider Man appears in nearly twice as many comic books as Mr. Fantastic and collaborates with a significantly wider cast of characters than Mr. Fantastic; however, Mr. Fantastic tends to appear in highly central comic books with large character casts. It is open to interpretation as to which measure of centrality is better, but in many applications, we tend to prefer CoHITS over HITS as CoHITS ranks are less influenced by the presence of outliers with extreme degrees (Aronson, Yang, Odabas, Ahn, & Perry, 2020).

It is also worth mentioning that assigning different edge weights to the network can significantly affect ranking results. Our package offers flexibility by allowing different combinations of algorithms and edge weights. We leave the choice to the users’ discretion.

Despite the ubiquity of bitpartite networks, bipartite PageRank algorithms are missing from the popular network packages, and our package serves to close this gap. Our target audience includes researchers and data scientists who deal with bipartite networks. To improve the accessibility, both R (birankr) and Python (birankpy) versions of the package are available. The documentation of BiRank consists of manual pages for its method functions, example usages, and unit tests.

## Figures and Tables

**Table 1:** A summary of transition matrices used in different BiRank algorithms. *K*_*T*_ and *K*_*B*_ are diagonal matrices with generalized degrees (sum of the edge weights) on the diagonal, i.e. ${\left(K_{T}\right)}_{ii}=\sum_{j}w_{ij}$ and ${\left(K_{B}\right)}_{jj}=\sum_{i}w_{ij}.w_{ij}$ is the element on row *i* and column *j* of the bipartite network adjacency matrix *W*^|*T*|×|*B*|^.

Transition matrix	*S*_*B*_	*S*_*T*_
HITS	*W* ^⊤^	*W*
Co-HITS	$W^{⊤}K_{T}^{-1}$	$WK_{B}^{-1}$
BGRM	$K_{B}^{-1}W^{⊤}K_{T}^{-1}$	$K_{T}^{-1}WK_{B}^{-1}$
Birank	$K_{B}^{-1/2}W^{⊤}K_{T}^{-1/2}$	$K_{T}^{-1/2}WK_{B}^{-1/2}$

**Table 2:** Top five characters in the Marvel Universe collaboration network ranked by HITS, CoHITS and PageRank with one-mode projection.

Rank	HITS	CoHITS	Projection+PageRank
1st	Captain America	Spider-man	Captain America
2nd	Iron man	Captain America	Spider-man
3rd	Thing	Iron man	Iron man
4th	Human torch	Hulk	Wolverine
5th	Mr. fantastic	Thing	Thor
